# Experiences of transitioning between settings of care from the perspectives of patients with advanced illness receiving specialist palliative care and their family caregivers: A qualitative interview study

**DOI:** 10.1177/02692163211043371

**Published:** 2021-09-03

**Authors:** Ping Guo, Cathryn Pinto, Beth Edwards, Sophie Pask, Alice Firth, Suzanne O’Brien, Fliss EM Murtagh

**Affiliations:** 1 School of Nursing, Institute of Clinical Sciences, College of Medical and Dental Sciences, University of Birmingham, Birmingham, UK; 2 Cicely Saunders Institute of Palliative Care, Policy & Rehabilitation, Florence Nightingale Faculty of Nursing, Midwifery & Palliative Care, King’s College London, London, UK; 3 Wolfson Palliative Care Research Centre, Hull York Medical School, University of Hull, Hull, UK

**Keywords:** Advanced illness, end of life care, experiences, palliative care, qualitative, transitions

## Abstract

**Background:**

Transitions between care settings (hospice, hospital and community) can be challenging for patients and family caregivers and are often an under-researched area of health care, including palliative care.

**Aim:**

To explore the experience of transitions between care settings for those receiving specialist palliative care.

**Design:**

Qualitative study using thematic analysis.

**Setting/participants:**

Semi-structured interviews were conducted with adult patients (*n* = 15) and family caregivers (*n* = 11) receiving specialist palliative care, who had undergone at least two transitions.

**Results:**

Four themes were identified. (1) Uncertainty about the new care setting. Most participants reported that lack of information about the new setting of care, and difficulties with access and availability of care in the new setting, added to feelings of uncertainty. (2) Biographical disruption. The transition to the new setting often resulted in changes to sense of independence and identity, and maintaining normality was a way to cope with this. (3) Importance of continuity of care. Continuity of care had an impact on feelings of safety in the new setting and influenced decisions about the transition. (4) Need for emotional and practical support. Most participants expressed a greater need for emotional and practical support, when transitioning to a new setting.

**Conclusions:**

Findings provide insights into how clinicians might better negotiate transitions for these patients and family caregivers, as well as improve patient outcomes. The complexity and diversity of transition experiences, particularly among patients and families from different ethnicities and cultural backgrounds, need to be further explored in future research.

**What is already known about the topic?**

- Transitions of care between settings can be challenging to negotiate for both patients and their family caregivers.
- Despite this, transitions are often an under-researched area of health care including palliative care. It has been suggested that transitions might be experienced differently by those with different palliative care conditions.
- We need to better understand the experience of transitions between care settings, to address the needs and concerns of patients with advanced illness and their family caregivers and offer appropriate support.

**What this paper adds**

- This study demonstrated that lack of information and poor communication added to feelings of uncertainty and was a potential stressor for both patients and family caregivers.
- Patients and family caregivers interviewed in this study adapted better to their transitions if they were able to redefine their view of what was normal and ‘carry on as normal’.
- Lack of continuity between care settings led to participants feeling unsafe and uncertain about transitioning to the new care setting.
- Family caregivers often act as advocates in the healthcare setting and play an important role not only in the practical realm, such as arranging transport or coordinating care, but also in providing emotional support.

**Implications for practice, theory or policy**

- Healthcare professionals should acknowledge that transitioning to a new care setting can be a stressful or difficult experience for patients and families, therefore, having options and being available to offer support is important when needed.
- Recommended changes to improve experiences of transitions included improving communication between teams and across organisations, clarifying accountability as patients move across settings, standardising discharge processes and providing additional in-house staff training for different health professionals on psychosocial support, communication skills and information giving.
- This study emphasises the importance of person-centred seamless care and demonstrates that better integration of palliative care in all settings is needed. Family needs assessment and support are crucial in transitions and caring for patients with advanced illness.
- Future research should explore the experiences of patients and families with a variety of ethnic and cultural backgrounds and with transitions from and to care homes, as every transition seems to be unique and context specific.

## Background

Transitions are defined as ‘significant life events that require a new situation or circumstance to be incorporated into a person’s life’.^1^ They are considered as a psychosocial process through which persons deal with significant developmental, organisational or situational changes.^2,3^ Patients with advanced illness and family caregivers may make transitions between different specialist palliative care settings, including between hospice inpatient, hospital inpatient, their home and community (e.g. nursing homes) settings. Transitions of care between settings can be challenging to negotiate for both patients and family caregivers. A narrative review of patient and family caregivers’ transition experiences of palliative care from the hospital to home setting showed that they experienced a broad range of needs during the transition process, including communication and information needs, emotional support and social support for family caregivers, which are not always addressed by healthcare professionals.^4^ The review indicated a need to further explore the experiences of patients and families, especially during the stage prior to the transfer from the hospital to the home.

Evidence suggests that being transitioned between settings can increase the risk of fragmented care from multiple healthcare providers and medical errors, which impede the provision of high-quality palliative care.^5–7^ There are only a few studies conducted on place of care and transitions between care settings in the final months of life and the extent to which needs and preferences of patients suffering from life-limiting diseases or their family caregivers play a role when the patients move between settings has not been well studied. A international mortality follow-back study of 4791 non-sudden deaths (2009–2011) demonstrated that end-of-life transitions between care settings are common across EU countries; 59%, 55%, 60% and 58% were transferred between settings at least once in the final 3 months of life in Belgium, Netherlands, Italy and Spain respectively, and 10%, 5%, 8% and 12% were transferred three times or more.^8^

A qualitative study from the UK interviewed 30 older adults with heart failure, lung cancer and stroke in the last year of life, and found that as these adults transitioned between care settings, much of the care received was characterised by inflexibility and a failure of healthcare professionals to listen. Liaison between and within services was not always effective, and community support after a hospital admission was perceived to be absent, inappropriate or excessive.^9^ A meta-synthesis review suggested that family caregivers experienced a ‘life transition’, whereby their lives were permanently altered when caring for community-dwelling persons with advanced cancer at the end of life. Programmes and interventions should be designed to support caregivers to ‘redefine normal’, and priority should be given to identifying ‘disruptions’ that may ultimately impact their ability to transition and maintain their role as caregivers.^10^

Palliative care providers struggle with the decision to transfer their patients to nursing homes.^11^ One qualitative study highlighted the tension palliative care physicians experienced when transferring patients with palliative care needs to a nursing home and the complexity of decision making. The process is often associated with feelings of abandonment and guilt in patients and families.^12^ It is imperative that discussions surrounding nursing home transfer are communicated and managed as sensitively as possible.^13^ Other evidence highlighted that patients wanted to receive information tailored to their own situation. A lack of continuity from secondary to primary care was also identified.^14^ As nonabandonment is one of clinicians’ central ethical obligations, information about goals of care and preferences should be clearly communicated during each of these transitions and the need for continuity of care and smooth transitions is critical in palliative care.^15^

Although there has been published literature on transition experiences in health care,^16^ it remains an under-researched area in palliative care. Transitions might have different meanings to people with different palliative care conditions, particularly those most vulnerable people with advanced illness who are experiencing these transitions often close to the end of their lives; whose voice is less often heard.^17^ Finding the best way to support patients with advanced illness and family caregivers as they experience transitions requires rich qualitative data. So far, qualitative studies have primarily contributed insight on what it is like living with incurable diseases and a particular type of transition (e.g. from the hospital to the home), but rarely focus on the experiences of transitions during advanced illness from both patients’ and family caregivers’ perspectives and various types of transitions across settings. Evidence has highlighted specific challenges in transitions, but more research is needed to understand what influences positive or negative transition experiences, and how these factors impact on patient outcomes. The present study strives to fill this gap and contributes to new knowledge by focussing on those with advanced illness at the very end of their lives, to shed light on the experiences of people who have transitioned between hospital, hospice and community settings, to identify if any different patterns of transition experiences exist, and to inform practice and policies in supporting patients and family caregivers when needed in transitions.

## Aim

To explore the experiences of transitions between care settings from the perspectives of patients with advanced illness receiving specialist palliative care and family caregivers, to understand what influences their transition experiences, and how transitions in care could be better negotiated to improve outcomes.

## Methods

Semi-structured, in-depth, interviews were conducted with patients and family caregivers receiving specialist palliative care, who were part of a larger longitudinal study. These types of interviews were utilised widely and extensively as interviewing format in health and social care research where the interview participants answered pre-set open-ended questions, with opportunities of allowing them to explore in-depth and delve from these questions.^18^ Data were collected on their experiences of care prior to, during and after the transitions between settings. ‘Transitions between care settings’ are – for this paper – defined as ‘a change in the place where a person receiving care is residing, between the person’s own home, a hospice inpatient unit, a hospital or a care home, any in any direction’. Usual place of residence may include hospitals, hospices and community settings. Consolidated criteria for reporting qualitative research (COREQ) were applied to improve transparency and credibility of this study.^19^

### Ethics

Ethical approval was granted by the NHS Health Research Authority London – Bromley Research Ethics Committee (16/LO/1021). All eligible patients and their main family caregivers were fully informed before consent was sought through the information sheets and verbal explanation on the aims and methods of the study and procedures that might be involved. Whatever they decided, patients’ care or legal rights were not affected in any way and even though participants gave consent for participation in the study, they could still change their mind at any time without giving any reason.

Our project Patient and Public Involvement (PPI) Advisory Group were consulted throughout the study to ensure that it was conducted in an ethical and respectful way and had the highest possible relevance and benefit to patients and families. If the participants became distressed during this study which raised concerns or warrant a change in their medical management, the predeveloped distress protocol was followed.

### Participants and settings

This qualitative study was embedded in a longitudinal mixed methods study (Reference: RP-PG-1210-12015) developing and testing a case-mix classification in palliative care in the UK.^20^ Participants in this qualitative study were adult patients receiving specialist palliative care and family caregivers, who had experienced at least two transitions between care settings. A purposive sampling approach was used to include participants from a range of age groups, genders, diagnoses, types of transitions in either direction and geographical areas to capture diverse perspectives relating to the experiences of transitions between care settings (Table 1).

**Table 1.** The proposed purposive sample selection.

*N*	Purposive categories				
Age group	18–45 years	46–65 years	66–75 years	76–85 years	86 year+
	2–5	5–8	5–8	5–8	5–8
Gender	Male	Female			
	8–12	8–12			
Diagnosis	Cancer	Non-cancer			
	12–16	4–8			
Type of transition	Hospital-home	Hospice-home	Hospital-hospice	Care home-hospice	Care home-hospital
	5	5	5	5	5
Patient lives alone	Living alone	Not living alone			
	8–12	8–12			
Geographical location	London	Dorset	Sussex	Staffordshire	Kent
	5	5	5	5	5

### Recruitment

Participants recruited to the longitudinal study were asked to indicate if they were interested in taking part in this qualitative study. Researchers then selected potential participants based on the purposive sampling criteria and asked them to confirm if they were willing to be interviewed about transitions of care. If the potential participants expressed their willingness to be interviewed, the researcher provided an information sheet and further introduced the study. A time and location convenient to the participant were arranged to obtain written consent and conduct the interview.

### Interview data collection

The interviews were conducted once only with the participants after at least two transitions between care settings and generally lasted the duration of 30–60 min. A topic guide was developed based on the study objectives and piloted with the project PPI members, and used to explore questions more systematically and comprehensively as well as to keep the interview focussed on the topic of our interest. All interviews were semi-structured and conducted by research assistants trained in interview skills (BE, CP, SO and SP). Participants were interviewed either at their home or in a quiet room where they received care (e.g. nursing home or hospice). Interviews were audio-recorded, and field notes were written. Some patients were interviewed on their own; others interviewed jointly with one family caregiver.

Interview questions in the topic guide were open-ended and comprised of the core question and some associated questions about the experience of transitioning between care settings; prompts included key issues identified in previous studies, including communication, coordination of care, information and support needs and discharge planning, to determine what worked well, what did not and where transitions could be improved. Data from the first five interviews were analysed and fed back to our PPI group and research team, for refinement of the topic guide to help probe for subsequent interviews. Data collection and analysis were conducted concurrently, with results of ongoing analysis informing continuing data collection. Data were collected until data saturation was reached when no new themes or categories emerged from interviews, and sufficient depth of understanding was achieved in relation to emergent theoretical categories.^21^

### Analysis

Audio-recordings were transcribed verbatim, checked for accuracy, anonymised and entered NVivo V12. Data were independently coded and analysed by two team members (BE and CP). BE developed the coding structure with assistance from CP until it captured all concepts about participants’ experiences of transitions in care. We adopted a similar approach to Pinnock et al.’s^17^ qualitative study, undertaking a thematic^22^ and narrative analysis of interviews, exploring how perspectives evolve over time, with detailed attention to patient and family perspectives on experience of care in each setting and transitions, including potential interventions to influence changes in care settings. BE and CP compared concepts within and across interviews, grouped similar concepts into themes and subthemes and refined the coding structure through discussion with PG and FM who read all transcripts. Themes and subthemes were then reviewed by the other authors to ensure consistency of interpretation and improve rigour of data analysis.

Since this qualitative study was embedded in a larger study, the prolonged engagement of the researchers helped to gain familiarity and understanding of the culture and context surrounding the participants being studied, which allowed the researchers to obtain more open and honest responses from participants.^23^ However, the researchers might be a source of bias during the data collection or analysis process as their point of view could have potentially influenced either participants’ responses or how those responses were interpreted. In addition, due to time constraints, each participant was not asked to verify the completeness and accuracy of an interview transcript, therefore, the project PPI members were consulted to improve data interpretation and ensure that the interpretation of findings truthfully reflected the meaning and intent of the participants’ contribution.

## Findings

20 interviews with 26 participants were conducted between 1st March 2017 and 31st May 2018. Participants’ ages ranged from 36 to 91 years (mean age = 68). Of these 20 interviews, 14 were conducted with patients and family caregivers separately and six jointly. The decision about whether the interviews were conducted individually or jointly depended on the participants’ preferences. Most interviews took place at the participant’s home (*n* = 17), hospice (*n* = 2) or care home (*n* = 1) where they were residing at the time of the interviews (Table 2). Interviews lasted from 12 min (cut short due to fatigue) to 95 min in duration (mean = 40 min). Four main themes and ten subthemes were identified (Table 3).

**Table 2.** Demographic characteristics of participants (*N* = 26).

Characteristics	*N* (%)
Age group	
18–45 years	3 (12)
46–65 years	4 (15)
66–75 years	13 (50)
76–85 years	5 (19)
86 year+	1 (4)
Gender	
Male	9 (35)
Female	17 (65)
Patient diagnosis (*n* = 20)	
Cancer	16 (80)
Non-cancer	4 (20)
Type of transitions between care settings	
Hospital to home	13 (25)
Home to hospital	14 (26)
Hospice to home	10 (19)
Home to hospice	10 (19)
Hospital to hospice	3 (6)
Hospice to hospital	0 (0)
Hospital to another hospital	2 (4)
Care home to hospice	0 (0)
Hospice to care home	1 (2)
Care home to hospital	0 (0)
Hospital to care home	0 (0)
Interview type (*n* = 20)	
With the patient only	9 (45)
With the caregiver only	5 (25)
Joint interview	6 (30)
Geographical location	
London	13 (50)
Dorset	5 (19)
Sussex	5 (19)
Staffordshire	2 (8)
Kent	1 (4)

**Table 3.** Themes and subthemes.

Themes	Subthemes
Uncertainty about the new care setting	Knowledge about the new care setting and discharge plan
	Access and availability of care
	Communication and information
Biographical disruption	Adjustment to identity change
	Maintaining normality
Importance of continuity of care	Tired of retelling stories
	Feeling unsafe
	Resisting transitions
Need for emotional and practical support	Support from family and friends
	Isolation

### Theme 1: Uncertainty about the new care setting

#### Knowledge about the new care setting and discharge plan

Most participants reported that the lack of information about the new setting they were being transferred to added to their feeling of uncertainty, and was therefore a potential source of stress:

*‘I was very, very nervous, didn’t know what to expect, and frightened. Because I wasn’t certain what goes on, what they do, who they all are, and it’s daunting when you don’t know anybody there already’.* (Interview 1, patient discussing a move from home to hospice)

Only one patient explicitly stated that he did know the new setting of care through a pamphlet provided by his family members:

*‘No, I wasn’t worried that the hospice didn’t provide me with information, because my nephew and niece were providing me with it. Indeed, they also showed me a pamphlet which described the place and showed photographs of the building and the inside of the building. Also, the facilities that were on offer’.* (Interview 9, patient discussing a move from hospice to nursing home)

People spoke of uncertainty regarding their discharge plan (e.g. when they would be discharged). Patients and families valued discussions early on, so that they could have enough time to prepare and get ready for transitions, although they acknowledged that the healthcare professionals might not have all the information needed.

*‘I don’t think you can even say lack of staff or anything else, it was lack of caring, it was lack of organisation. And just basically, you know, I would have liked to have had a talk to people about, ‘What happens to me now?’ (Laughter) Because what do you do? Go home? I don’t know if you had to do anything, and stuff like that’.* (Interview 12, patient discussing a move from hospital to home)

Some participants emphasised the practical and logistical frustrations like delays.

*‘They just could not say when they were coming. Once you are discharged from your room, you are sitting really waiting. . .. . . then you are just like anywhere. You are looking for – if it is too long you are waiting’.* (Interview 13, jointly discussing a move from hospice to home)

#### Access and availability of care

Many participants commented on ‘bad’ timing of a transition (e.g. emergency admissions during out of hours) and uncertainty about access to care. This included identifying the correct point of contact and which team is responsible for different aspects of care, particularly when urgent problems arose in the middle of the nights or during weekends.

*‘The thing we didn’t realise, because by this time it was Sunday morning, it’s a weekend, there’s no oncology department at this particular weekend, so you were basically in a holding position until [husband] could be admitted back to hospital’.* (Interview 8, jointly discussing a move from home to hospital)

Uncertainty about the availability of beds and adequate medical support in the new setting remained as a concern. Participants discussed the NHS winter crisis, which resulted in many patients waiting in corridors for beds and treatment in a stretched public hospital system.

*‘Again, they were overstretched, not to the same extent that [hospital 1] were, but they were overstretched and things. We just thought, “This isn’t-” and medically-wise, there wasn’t really anything else they were going to do. So, it didn’t make any sense, so we did push for her to go to [hospice]’.* (Interview 15, carer discussing a move from hospital to hospice)

#### Communication and information

Participants highlighted that clear and effective communication is as important as the quality of care delivered. They considered it critical to find a common language that could be understood and spoken by both sides.

*‘I also had conversations with the same consultant. . . He was very clear, very communicative. . . I think he was just a very good communicator as well as a good doctor. The two together – brilliant’.* (Interview 8, jointly discussing care in a hospice)

Most participants felt there was good communication between staff within multidisciplinary teams and across different teams, and they were treated with empathy, humanity and kindness. However, one patient indicated that staff should communicate with patients and their families in a more private and sensitive manner to avoid additional distress, particularly when bringing ‘bad’ news to them.

*‘The lady opposite me, who was very, very poorly, and who I’d been talking to, she actually started to cry. So, this was distressing, to have the woman opposite me crying, and the old lady next to me, there was a curtain between us, but the old lady next to me was going, ‘(Gasping sound),’ every time they said anything. And I was just sitting there like a zombie. It was like, ‘What?’ Anyway, so [doctor] said, ‘You have grade 4 bowel cancer, and you have secondaries . . .’’* (Interview 12, patient discussing care in a hospital)

Participants found that receiving the right information in the right place at the right time helped them to gain a better understanding of health status and care plan, which would reduce their anxiety and worries. However, some reported that they had not received enough information. Family members felt they were not being informed about the situation of their loved one after a transition.

*‘They put me in the relatives’ room, which I must say, is like sitting in a cell. It’s not ideal, but circumstances are they are, and you take what’s offered. But there was nowhere to get a drink, because the machine was broken. Nobody came out and gave me any information’.* (Interview 6, jointly discussing care in a hospital)

### Theme 2: Biographical disruption

#### Adjustment to identity change

Sense of identity was considered important to people, but participants felt that transitions often compromised one’s sense of self, which linked to inevitable loss of control and independence. Patients and caregivers tended to adapt better to their transitions if they were able to accept identity change. As a female patient says:

*‘You’re living with a different body, and you’re living with a different mind-set, having thought you were going to probably live until you’re well into your 80s and stuff like that. You’re living with all these different things and you’re on drugs and stuff that are changing just your everyday stuff. And there has got to be some understanding of that’.* (Interview 12, patient discussing a move from hospital to home)

For both patients and caregivers, changes in needs and treatment after transitions require periods of adjustment and news ways of coping. They worried about physical decline but were adapting their new roles and identities.

*‘I suppose the fundamental difference is two-fold. One is my physical condition, that unfortunately I am now trapped in a wheelchair. I can’t stand upright, and I can’t walk. I am undergoing physiotherapy classes and they are beneficial, undoubtedly. I am gaining confidence and strength, but that is one of the facts of my life now’.* (Interview 9, patient discussing a move from hospice to nursing home)

#### Maintaining normality

There was recognition that patients and families trying to maintain a sense of normality becomes important when everything else is changing. Knowing oneself was a source of strength and security and a buffer against detrimental consequences of transitions.

*‘I said to my husband, ‘you are not going to be like [sister]. We’re going to carry on as normal once you get better,’ and we have done’.* (Interview 3, carer discussing a move from hospice to home)

### Theme 3: Importance of continuity of care

#### Tired of retelling stories

Both family and patients praised clinicians who provided continuity and got to know them as individuals. They appreciated not having to retell their stories and recognised the difference in care when staff knew them.

*‘Now, [name of service] was sending me someone to get me up in the morning for 45 minutes and it was a different person every day. By the time I had explained to her how this will work, there was no time left for washing, dressing or breakfast. . .. Why can’t [name of service] arrange it so that you have some sort of, as you say, continuity? Continuity when you’re ill is really important’.* (Interview 7, patient discussing care at home)

#### Feeling unsafe

Participants highlighted the importance of the correct and timely transfer of patient information, medical notes and medication prescriptions when moving across settings within one institution or across different institutions. They expressed that when things were disjointed and staff members were not fully aware of the circumstances, patients and their families felt unsafe, and lack of continuity could lead to medical mistakes.

*‘Anyway, that got sorted out eventually, but it makes you nervous. That’s when you don’t feel safe when there are people dealing with you who don’t know your history, who clearly haven’t read the notes and who are prescribing things that you think are not what they should be doing’.* (Interview 7, patient discussing care in a hospital)

#### Resisting transitions

Most participants emphasised that to keep continuity of care, they would prefer to continue staying in their current setting and not move between settings unless necessary.

*‘. . . when my husband was taken ill, it was a choice of going into the hospice or trying to stay at home with care at home. Although the preferred idea was to go into the hospice, because there is so much to do for me now and it is so easy for me with the carers that know what they are doing, I did ask specifically, if possible, to stay at home’.* (Interview 13, jointly discussing a move from home to hospice)

*‘No. I didn’t want to go. I fought against it. Because I go in and out with the same thing really. Similar thing. And I know that they keep you in’.* (Interview 5, patient discussing a move from home to hospital)

### Theme 4: Need for emotional and practical support

#### Support from family and friends

Family members often act as advocates in the healthcare setting and most participants specified a great need for support from family and friends not only in the practical realm, such as arranging transport or coordinating care but also in the emotional aspect.

*‘God, you’ve got to have family. I don’t care what you say. You’ve got to have family behind you. I thought I could stick up for myself, but you need your family as well. And there are a lot in there that haven’t got it’.* (Interview 5, patient discussing a move from hospital to home)

#### Isolation

For those patients who lived alone and did not have a family caregiver to help them, their experience of transitions was more challenging. Some commented that there was no one to drive them home from the hospital or hospice and stated that they had to rely on hospital transport, which was often unreliable or inflexible and sometimes required a long wait. Others talked about their experiences of being alone and felt being neglected in care.

*‘And as I was sitting there on my own, I said, ‘Excuse me, doctor,’ I sort of said, ‘My son works half an hour from here,’ and I said, ‘I have friends,’ you know? Like, ‘I’m a person,’ (Laughter) do you know what I mean? And I should have been asked, ‘Would I like someone to be with me?’ Or for that matter, even a nurse. Do you know? But nobody’.* (Interview 12, patient discussing a move from hospital to home)

In addition, some participants spoke more positively about their experiences of care in hospices than in hospitals. Lack of emotional support and information received from hospital settings were considered as major issues, which should be urgently addressed to improve patient care and experiences. There were no patterns found from our data to demonstrate the differences between different types of transitions. However, the transitions that worked better tended to be those during which relevant staff members communicated and coordinated well between teams and between organisations to put the patients’ needs and preferences at the centre of care, to ensure safe and seamless transitions.

## Discussion

### Main findings

This study has explored the experiences of transitions between care settings among patients with advanced illness receiving specialist palliative care and family caregivers and identified the challenges faced prior to, during and after a transition. Our findings identified the importance of communication and information giving, recognition and acceptance of identity change, continuity of care and provision of emotional and practical support that contributed to their overall experiences of transition and provided implications on how transitions in care could be better negotiated.

Patients often experience unavoidable transitions from one health care setting to another in the last months of life, which can become a source of stress for both patients and their families, especially in the absence of information and lack of communication.^9,24^ Fear and uncertainty of a new place they were transferring to was recognised by the patients and family caregivers in our study as the most common experiences of transitions and they wanted more information about the new care setting prior to transfer. An integrative review highlighted that patient and family engagement in communication with health professionals during transitions of care to, within and from acute care settings could benefit all, and organisational strategies to improve information-giving and communication about transitions must incorporate an understanding of patient needs.^25^ Our findings reconfirmed that information and communication needs are important to ensure that patients and their families know what to expect from their transition and this aspect of care should be improved no matter which setting the patients currently stayed in (including hospital, hospice or community-based settings). In addition, communication training is required for health care professionals to respect their patients’ right to be informed and health care professionals can adopt a context sensitive approach to considering the timing, privacy, location and appropriateness for patients and families when communicating with them.

Patients and family caregivers interviewed in this study seemed to better adapt to their transitions if they were able to accept identity change, redefine their view of what was normal and ‘carry on as normal’. They found it easier to maintain their sense of self, feel in control and confident in coping with challenges and find hope and meaning in their new lives. Our finding is consistent to Selder’s^3^ life transition theory and other transition theories^2,26,27^ in which the importance of integrating transitions into a person’s life through a process of normalisation is necessary. A qualitative study exploring influences on the care preferences of frail older people with recent acute illness highlighted that most participants wanted to achieve a sense of normality in their daily lives, and normality could be achieved either by ‘getting back to normal’ or, where this was not possible, by ‘finding a new normal’.^28^

Continuity of care is critical in healthcare delivery and can improve patient outcomes such as higher patient satisfaction with medical care and lower hospitalisation rates.^29^ However, linking the care components into a coherent trajectory including a smooth transition process can be challenging and requires better coordination of care between services.^30^ Patients with advanced progressive conditions, their family caregivers and healthcare professionals identified poor care coordination at transitions for patients in the last year of life, particularly during emergency admissions and discharge.^31^ This is consistent with our findings which suggested that the participants felt unsafe and uncertain about transitioning to a new care setting, partly due to a lack of continuity of care. A meta-synthesis of qualitative studies also showed that communication and information transfer across care settings as well as the gathering of holistic information about the patient were perceived crucial by patients to foster continuity, and patients considered their personal involvement was one facilitating element of continuity of care.^32^ Ambiguity about who is being handed off to and time pressures in the acute setting may lead inpatient providers to give lower priority to discharge communication.^33^ Involving the patients and caregivers in goal setting, informing their transitions in care and providing timely communication have been recommended as effective approaches to improve continuity and transition experiences specific to persons living with dementia.^34^

### Implications for future practice

Poor communication across care settings can lead to chaotic, unsystematic transitions, poor patient outcomes and feelings of futility and dissatisfaction among providers. Patients with complex psychosocial needs were especially vulnerable and faced the challenges of coping with uncertainty during transitions. Recommended changes to improve experiences of transitions included improving communication between teams and across organisations, clarifying accountability as patients move across settings, standardising discharge processes and providing additional in-house staff training for health professionals on psychosocial support, communication skills and information giving.^35^

People prefer to die at home, and dying in usual place of residence is often described as an important indicator for the quality of palliative and end of life care.^36–38^ In reality, many patients are hospitalised in the last months of life^39^ and there has been a mismatch between preferences for place of death and actual site of death.^40^ Frequent transitions between care settings in palliative and end of life care can be associated with an increased risk of fragmented care and exposure to unnecessary treatments and medical errors.^41,42^ However, an international study across four European countries (Belgium, the Netherlands, Italy and Spain) demonstrated that over 50% of patients had at least one transition in care settings in the last 3 months of life; one third of patients in Belgium, Italy and Spain had a last week hospital admission and died in hospital. The Netherlands had less frequent transitions, which could be attributed to the diverse models of health care and the role of General Practitioners as the main providers and referents for palliative care.^43^ Another study conducted in Italy suggested that among cancer decedents having three or more than three transitions in the last month, the most common care trajectories were home-hospital-home-hospital (36%), home-hospital-home-hospice (13%), hospital-home-hospital-hospice (12%) and hospital-home-hospital-home (9%).^44^ The transitions between care settings experienced at end of life by the patients with advanced illness and family caregivers in our study indicated the importance of person-centred seamless care and emphasised that further integration of palliative care into all care settings is required across the globe.

Transitions are vulnerable times not only for patients, but also for their families, which may be associated with increased family stress. The complexity of the discharge process and disjointed nature of the health care system can further complicate transitions. Madsen et al.^45^ explored patients’ experiences of transitions during courses of incurable cancer and found that some patients experienced their family members distancing themselves from them, which was a major change in their lives and left them feeling isolated. This could be explained by excessive anxiety and stress family members experienced due to burdensome caring responsibilities including transitions. In our study, role of family was identified as critical to successful transitions, which could lead to optimal patient outcomes post-discharge such as reduced hospital readmission. This is consistent with findings from a qualitative study which suggested that transitions from hospital to home affect the lives of families in ways which may affect patient outcomes post-discharge.^46^ Family needs assessment and support are crucial in transitions and caring for patients with advanced illness.

### Strengths and limitations

This study deepens understanding of the transition experience of patients with advanced illness and family caregivers. It not only provides recommendations of how transitions in care could be better negotiated to improve outcomes, but also emphasises the importance of person-centred care and continuity, which implies that better integration of palliative care across settings is needed.

There are several limitations to this study. Patients and families who were excluded from this study (e.g. those lacking capacity due to cognitive impairment or neurological conditions) or declined to participate may have different experiences of transitions. Although we tried to ensure that participants from a range of age groups, genders, diagnoses and geographical areas were included, participants were all white British from the UK except one from Australia, and experiences from other ethnicities may not be represented in the findings. Most participants (80%) had cancer, which is consistent with the findings from a multisite mixed methods study of patients referred for specialist palliative care suggesting that the majority of patients receiving specialist palliative care (87%) had a primary diagnosis of cancer.^47^ Therefore, the experiences of transitions among patients with non-cancer conditions needs further exploration. The lack of representation in transitions from and to care homes has limited the ability to explore and compare the perspectives of people having transition experiences of care homes with other types of transitions. Future research should explore the experiences of patients and families with a variety of ethnic and cultural backgrounds and with transition experiences of care homes as every transition seems to be unique and context specific.

## Conclusions

Transitions are often a time of uncertainty; this needs to be mitigated by effective communication and information giving, recognising the importance of identity, continuity of care and emotional and practical support. The diversity and complexity of transition experiences particularly among patients and families from different ethnicities and cultural backgrounds needs further research.
